# Surgical resection of lung adenocarcinoma after crizotinib treatment: report of a case

**DOI:** 10.1186/s12957-015-0480-2

**Published:** 2015-02-22

**Authors:** Kaoru Kaseda, Ken-ichi Watanabe, Keisuke Asakura, Akio Kazama

**Affiliations:** Department of Thoracic Surgery, Sagamihara Kyodo Hospital, 2-8-18 Hashimoto, Midori-ku, Sagamihara, Kanagawa 252-5188 Japan; Department of Pathology, Sagamihara Kyodo Hospital, 2-8-18 Hashimoto, Midori-ku, Sagamihara, Kanagawa 252-5188 Japan

**Keywords:** Salvage surgery, Crizotinib, Anaplastic lymphoma kinase, Non-small cell lung cancer

## Abstract

A 45-year-old female was diagnosed as having lung adenocarcinoma harboring an anaplastic lymphoma kinase (ALK) rearrangement, stage IV (T2bN3M1b). She was treated with crizotinib as second-line chemotherapy. The clinical stage after crizotinib treatment was ycT2aN0M0, stage IB. We performed a left lower lobectomy and lymph node dissection aimed at local control and pathological confirmation of the remaining tumor. The final pathological stage was ypT2aN2M0, stage IIIA with Ef 1b. To the best of our knowledge, this is the first case report of surgical resection in ALK rearrangement-positive lung adenocarcinoma after crizotinib treatment.

## Background

Rearrangements of the ALK gene are present in 3% to 5% of non-small cell lung cancers (NSCLCs) [1,2]. They define a distinct subgroup of NSCLC that typically occurs in younger patients who have never smoked or have a history of light smoking and that has adenocarcinoma histologic characteristics [3-5].

Crizotinib is an oral small-molecule tyrosine kinase inhibitor (TKI) of ALK, MET, and ROS1 kinases [6]. Crizotinib competes with adenosine triphosphate for binding to the tyrosine kinase pocket of ALK and thereby inhibits its tyrosine kinase activity, leading to inhibition of downstream signaling and to anticancer effects.

In phase 1 and 2 studies, crizotinib treatment resulted in objective tumor responses in approximately 60% of patients with ALK-positive NSCLC and in progression-free survival of 7 to 10 months [7-9]. In a randomized phase 3 trial involving patients with advanced ALK-positive NSCLC who had received previous platinum-based chemotherapy, crizotinib was superior to single-agent second-line chemotherapy with either pemetrexed or docetaxel [10]. And also in a randomized phase 3 trial involving patients with previously untreated advanced ALK-positive NSCLC, crizotinib was superior to standard first-line pemetrexed-plus-platinum chemotherapy [11].

However, there has been no data about the preoperative crizotinib treatment in NSCLC patients. We report the case of a 45-year-old female who underwent surgical resection after crizotinib treatment in stage IV NSCLC.

## Case presentation

A 45-year-old female, current smoker with no significant medical history, was found to have a left lung mass in July 2012. Chest computed tomography (CT) revealed a 58-mm-diameter mass in the left lower lobe and bilateral hilar and mediastinal lymphadenopathy. Positron emission tomography (PET)-CT showed FDG accumulation in the lesions mentioned above and in the left axillary lymph node. The serum carcinoembryonic antigen (CEA) level was high as 71.0 ng/ml. Transbronchial biopsy of the primary lesion revealed adenocarcinoma. Head magnetic resonance imaging (MRI) was negative for metastases. The clinical stage was determined based on the *TNM Classification of the International Union Against Cancer (UICC), 7th edition*, and the patient was considered to have inoperable T2bN3M1b. stage IV disease. We evaluated the responsiveness of tumor to chemotherapy, based on Response Evaluation Criteria in Solid Tumors (RECIST), version 1.1.

**Figure 1:**
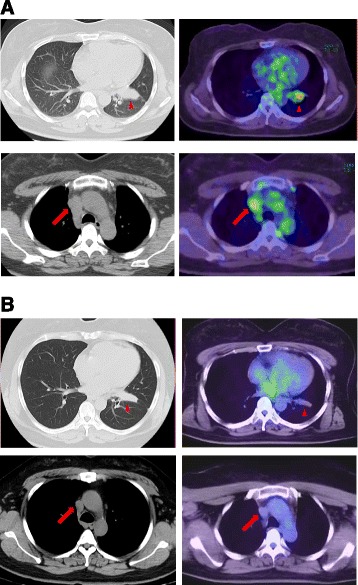
**Chest CT and PET-CT images before and after crizotinib treatment. (A)** Chest CT and PET-CT before treatment revealing a large primary lung mass (*arrowhead*) and multiple lymph node metastases affecting mediastinal and bilateral hilar lymph nodes (*arrow*). **(B)** Chest CT and PET-CT 6 months after initiation of crizotinib therapy showing significantly decreased tumor and no FDG accumulation in the primary and other lesions.

In August 2012, chemotherapy with cisplatin and pemetrexed was initiated, resulting in stable disease after four courses, followed by seven cycles of pemetrexed continuation maintenance therapy. Although she achieved stable disease for approximately 9 months, cancer regrowth occurred at the right mediastinal lymph nodes (Figure 1A).

**Figure 2:**
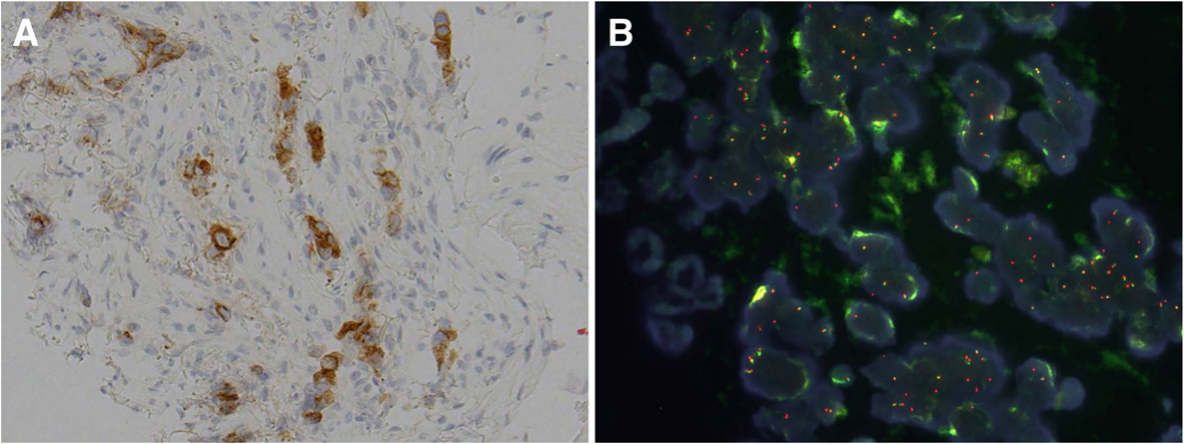
**Representative images of immunohistochemical staining and fluorescent** ***in situ*** **hybridization for ALK. (A)** Immunohistochemistry showed moderate ALK protein expression. **(B)** Fluorescent *in situ* hybridization analysis showed translocation of the ALK gene.

Immunohistochemistry (IHC) and fluorescent *in situ* hybridization (FISH) revealed that the tumor had an ALK rearrangement (Figure 2A,B). Crizotinib (250 mg twice daily), started in June 2013, produced a favorable response; chest CT showed marked decreases of the primary lesion and lymph nodes. PET-CT showed no FDG accumulation in the primary and other lesions (Figure 1B). A partial response was achieved and maintained for 6 months by crizotinib treatment. The clinical stage after crizotinib treatment was ycT2aN0M0, stage IB: 1) the serum CEA level decreased to 7.4 ng/ml once and increased slightly afterwards, 2) there is a possibility of tumor progression because of progression-free survival of crizotinib treatment, and 3) the patient’s hope for surgical resection of residual tumor was the reason for considering salvage surgery. Surgery for local control and pathological confirmation of the remaining tumor was performed with the patient’s consent. In December 2013, a left lower lobectomy and lymph node dissection were performed. Intraoperative findings showed rigid fibrosis surrounding the hilum of the lower lobe, considered to be a scar of a metastatic lymph node after crizotinib treatment. The operation took 4 h and 23 min, with blood loss of 200 ml. No blood transfusion was needed. The final pathological stage was ypT2aN2M0, stage IIIA with Ef 1b. The postoperative course was uneventful, and the patient was discharged on the eight postoperative day. The patient was free of disease 6 months after surgery.

## Discussion

Recently, several authors reported surgical treatment, which can be performed for local control and diagnostic intent after epidermal growth factor receptor (EGFR)-TKI gefitinib administration, showing long-term survival in some patients [12,13]. And the strategy of salvage surgery for super responder of targeted therapy seemed to be worth exploring.

Crizotinib is an oral small-molecule TKI targeting ALK. The present patient with ALK rearrangement-positive NSCLC showed pronounced and rapid regression of tumor only 6 months after starting crizotinib. Despite the remarkable downstaging after crizotinib treatment, the patient had a more advanced pathological stage than the preoperative clinical stage. Dramatic radiologic response does not necessarily correlate with cell death. The present result suggests initially expressed systemic disease was essentially unchanged even after dramatic radiologic response to crizotinib. Stage IV lung cancer is considered to be a systemic disease; therefore, surgical treatment is rarely viewed as a feasible option. Nevertheless, some reports indicate near-complete pathological response of advanced NSCLC after induction chemotherapy [12,14]. Systemic preoperative therapy is increasingly used in advanced NSCLC in order to downstage the disease, reduce the burden of distant micrometastases, and thus provide an opportunity for potentially curative resection. To the best of our knowledge, this is the first case report of surgical resection in ALK rearrangement-positive lung adenocarcinoma after crizotinib treatment.

## Conclusions

We report a case of surgical resection of lung adenocarcinoma after crizotinib treatment. The present report provides insight into the efficacy of surgery after crizotinib therapy, but a prospective study is needed to verify this and the optimal duration of ALK-TKI treatment, the timing of surgery, and the role of adjuvant ALK-TKI treatment.

## Consent

Written informed consent was obtained from the patient for the publication of this case presentation and accompanying images. A copy of the written consent is available for the review by the Editor-in-Chief of this journal.

## Abbreviations

ALK: anaplastic lymphoma kinase
CEA: carcinoembryonic antigen
CT: computed tomography
EGFR: epidermal growth factor receptor
EML4: echinoderm microtubule-associated protein-like 4
FISH: fluorescent *in situ* hybridization
IHC: immunohistochemistry
MRI: magnetic resonance imaging
NSCLC: non-small cell lung cancer
PET: positron emission tomography
TKI: tyrosine kinase inhibitor
